# Towards the Development of a Smart Flying Sensor: Illustration in the Field of Precision Agriculture

**DOI:** 10.3390/s150716688

**Published:** 2015-07-10

**Authors:** Andres Hernandez, Harold Murcia, Cosmin Copot, Robin De Keyser

**Affiliations:** Department of Electrical Energy, Systems and Automation (EeSA), Ghent University, 9000 Ghent, Belgium; E-Mails: harold.murcia@unibague.edu.co (H.M.); cosmin.copot@ugent.be (C.C.); Robain.DeKeyser@ugent.be (R.D.K.)

**Keywords:** UAV, remote sensor, precision agriculture

## Abstract

Sensing is an important element to quantify productivity, product quality and to make decisions. Applications, such as mapping, surveillance, exploration and precision agriculture, require a reliable platform for remote sensing. This paper presents the first steps towards the development of a smart flying sensor based on an unmanned aerial vehicle (UAV). The concept of smart remote sensing is illustrated and its performance tested for the task of mapping the volume of grain inside a trailer during forage harvesting. Novelty lies in: (1) the development of a position-estimation method with time delay compensation based on inertial measurement unit (IMU) sensors and image processing; (2) a method to build a 3D map using information obtained from a regular camera; and (3) the design and implementation of a path-following control algorithm using model predictive control (MPC). Experimental results on a lab-scale system validate the effectiveness of the proposed methodology.

## 1. Introduction

In the last few years, there has been great interest from different industries to obtain better product quality at higher production rates, to improve energy efficiency, while decreasing production costs. An essential element in achieving these goals is sensing; without reliable and accurate measurements, it is impossible to quantify productivity and, therefore, unfeasible to make timely corrections.

Depending on the application, different instrumentation structures can be found, but with a clear trend towards the use of multiple sensors, in order to collect all possible information about the system. Therefore, typically, sensor networks are used [1]. This is especially true for applications, such as production machines, automation, mapping, precision agriculture and weather forecasting, where a large area needs to be covered [2].

Sensing of large areas leads to difficulties, such as power supply, calibration procedures, data delays, accessibility issues for installation and maintenance, as well as high costs. Researchers have been working on developing wireless sensor networks to mitigate some of these problems [3]. A possibly more effective solution consists of using a remote sensor to ‘freely’ move in the space, thus increasing flexibility while diminishing costs, because that solution requires less sensors and is easier to maintain. In this matter, the use of aerial vehicles appears as an interesting option, since they have the ability to maneuver through complex environments. Even more interesting is the use of autonomous unmanned aerial vehicles (UAV) that can execute complex tasks without sustained human supervision, given their capability to perform tasks that are difficult or costly for manned aerial vehicles to accomplish [4]. In order to undertake the challenging task of automated flight and maneuvering, a versatile flight control design is required. One of the aerial vehicles that can accomplish this is a quadrotor due to its relatively small size, ability to hover and mechanical simplicity [5].

A large number of papers have emerged in the literature on quadrotors. Modeling, identification and control of a quadrotor are described by [6] using on-board sensing. Furthermore, simultaneous localization and mapping (SLAM) was implemented to create 3D maps of the environment, as well as to establish the quadrotor position in space [7]. Automatic navigation and object recognition with filtered data from on-board sensors and cameras is reported in [8]. Complex tasks, such as catching a falling object using a single quadrotor, have been accomplished in [5] or for a group of quadrotors in cooperative formation in [9], where high-speed external cameras were used to estimate the position of both the object and UAV. Difficult tasks, such as flying in cities or forests, require further progress on image processing, to achieve reliable detection of obstacles using low-weight hardware and low computational costs [10]. Current implementations in UAVs still require an on-ground operator with visual contact to the aerial vehicle for tasks, like taking off, landing, collision avoidance and adaptive path-planing. There exists a need to design methodologies to cope with these conditions in order to increase the degree of intelligence and therefore autonomy of UAVs.

Regarding applications for remote sensing, precision agriculture, exploration, surveillance and mapping are some of the main activities, because a smart remote sensor can be useful to take information from different angles and/or follow predefined paths in areas that are difficult access for human beings [11,12]. For example, in the field of precision agriculture, the goal is to gather and analyze information about the variability of soil and crop conditions, in order to maximize the efficiency of crop inputs within small areas of the farm field [13]. This requires obtaining reliable information about the crop conditions in real time. A possible solution might be to install a net of ground sensors; unfortunately, this is a costly and difficult to maintain solution. Mapping is also of high interest in activities such as cartography, archeology and architecture [14]. Here, the challenge lies in the accessibility to remote areas for a human being. In the case of exploration and surveillance, a smart remote sensor could autonomously fly above a pipeline of gas or petrol, in order to find leaks or detect corrosion, once the UAV is equipped with the necessary instrumentation.

This paper presents the first steps towards the development of a smart flying sensor, using as a flying platform a low-cost quadrotor. The task of mapping the volume of grain inside a trailer during forage harvesting is used as a test case to demonstrate the concept of smart remote sensing and to assess the performance of the proposed solution. In the context of this research, smart is linked to autonomy or the capacity of the flying sensor to make decisions without human intervention. Thus, automatic detection of the trailer, precise position control to avoid the phantom effect on the pictures and image processing algorithms to automatically generate a 3D profile are some of the features that make this a smart sensor. The main contributions are the development of: (i) a position-estimation method with delay compensation based on sensor fusion; (ii) a method to build a 3D map using information obtained from a regular camera; and (iii) a path-following predictive control algorithm to guarantee the accurate position of the UAV in space.

The paper is structured as follows. A description of the hardware, instrumentation and position estimation using sensor fusion is presented in Section 2. In Section 3, the quadrotor's dynamics and modeling is presented, followed by the path-following control algorithm in Section 4. Next, the effectiveness of the proposed smart remote sensor is tested for the task of mapping in a precision agriculture application, as described in Section 5. The final section summarizes the main outcome of this contribution and presents the next challenges.

## 2. Quadrotor Characteristics and Sensory Equipment

Quadrotors have four rotating blades, which enable flight similar to that of a helicopter. Movement is attained by varying the speeds of each blade, thereby creating different thrust forces. Today, they are equipped with on-board sensory equipment and the ability to communicate wirelessly with a command station, thus making it possible to implement advanced control algorithms to achieve precise control, even during aggressive aerial maneuvers.

In this work, the commercially available and low-cost AR.Drone 2.0 is used as the flying platform. A description of its main characteristics, sensory equipment and position estimation using sensor fusion is presented in this section.

### 2.1. Characteristics of the AR.Drone 2.0 Quadrotor

The quadrotor comes with internal in-flight controllers and emergency features, making it stable and safe to fly [15]. The only downside would be that access to the quadrotor's internal controller is restricted. The internal software is a black-box system, and the parameters that refer to control and other calibration procedures are not fully documented. There are four brushless DC motors powered with 14.5 W each, from the three-element 1500-mA/H LiPo rechargeable battery that provides an approximate flight autonomy of 15–20 min. Two video cameras are mounted on the central hull, pointing to the front and to the bottom of the quadrotor.

**Figure 1:**
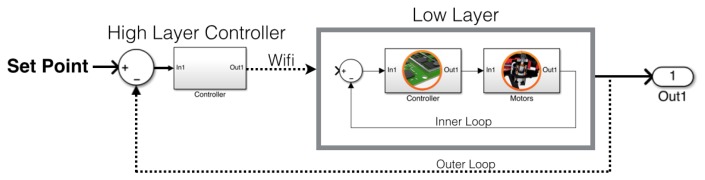
Quadrotor layers: the low layer represents the electronic assistance and the embedded operative system on the AR.Drone; the high layer represents the pilot (natively a smart device *i.e.*, iPhone).

This on-board black-box system in the AR.Drone 2.0 can be considered the low layer. The high layer is represented by the command station, which defines the references to the internal controllers located in the low layer. A schematic representation is depicted in Figure 1.

In this application, the high layer consists of a C++ application in Visual Studio, which allows accessing all AR.Drone communication channels, therefore enabling functions to send commands or set configurations, receive and store data from sensors and video-stream. Thus, data can be interpreted off- or on-line for modeling, identification or control purposes. Movement in the quadrotor is achieved by furnishing reference values as input to the internal black-box controllers.

### 2.2. Technical Specifications and Sensory Equipment

#### 2.2.1. Inertial Measurement Unit Board

The micro-electro-mechanical systems (MEMS)-based sensors are located below the central hull. They consist of:

a three-axis accelerometer of ±50 mg precision
a three-axis gyroscope with 2000°/s precision
a three-axis magnetometer of 6° precision

which together form the inertial measurement unit (IMU). The IMU provides the software with pitch, roll and yaw angle measurements. The measurements are also used for internal closed-loop stabilizing control (black-box).

#### 2.2.2. Ultrasonic Sensor

**Figure 2:**
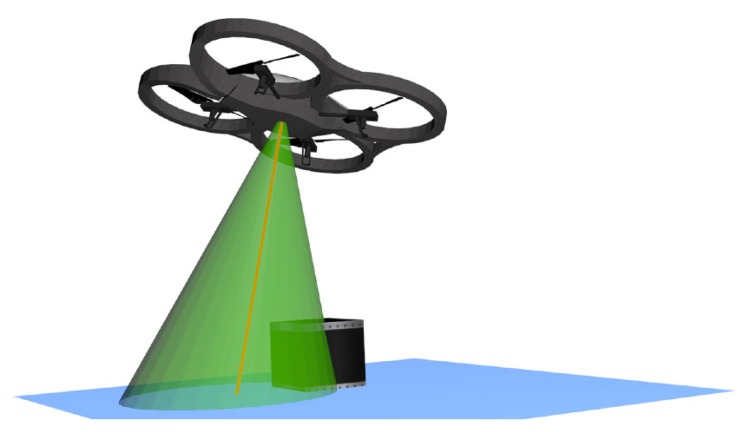
Overview of the ultrasound sensor cone: the green cone indicates the range of the ultrasound sensor [7].

The ultrasonic sensor is used for low altitudes (below 3 m); it operates using two different frequencies, 22.22 Hz and 25 Hz, in order to reduce noise or perturbations between quadrotors. It is important to clarify that even if the sound propagates in a cone-like manner, the ultrasonic sensor does not provide a map of what is inside the cone's area. Instead, it outputs a single measured value, which corresponds to the highest object on the ground present somewhere within the cone's area, as illustrated in Figure 2. This effect makes it more difficult to accurately determine the altitude in rough terrain or the presence of ground obstacles. The relation between the altitude and the diameter of the cone is represented in Equation (1).

(1) $$D_{\text{cone}}[m]=2*\tan(0.2182*\text{altitude}[m])$$

#### 2.2.3. On-Board Cameras and Calibration Procedure

The AR.Drone 2.0 has two cameras. The bottom camera, which uses a CMOS sensor, has an image size of 320 × 240 pixels. It is the fastest camera with a speed of 60 FPS and an angle of vision of approximately 64°. The frontal camera can be used for difference sizes: 1280 × 720 pixels or 640 × 360 pixels; the speed is 30 FPS, and the angle of vision is 80–90°, approximately; this big angle of vision is responsible for the radial distortion in the images, similar to a fish-eye effect.

**Figure 3:**
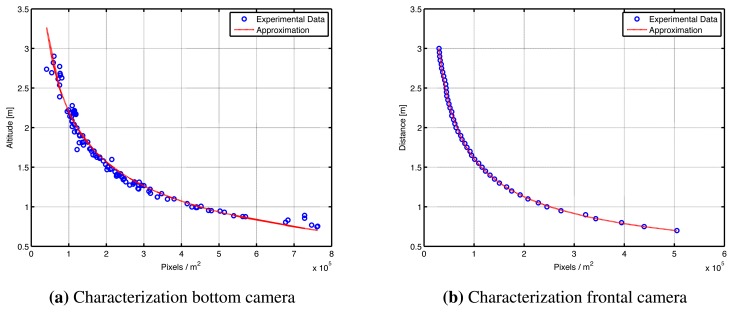
Camera characterization. Experimental data and approximation obtained for the (**a**) bottom and (**b**) frontal camera.

The cameras represent the main source of information for the system. Therefore, it is important to characterize the camera experimentally, in order to define the relation between pixels and meters. The experiment consists of taking pictures of a reference object of known characteristics (*i.e.*, height, width and color) at different distances. A fitting procedure is performed in order to characterize the cameras using the experimental as depicted in Figure 3, thus obtaining a relation which allows to compute the distance between the quadrotor and a reference object using Equations (2) and (3).

(2) $$\text{Altitude}[m]=148.6{\left(\frac{\text{Pixels}}{\text{Area}}\right)}^{-0.339}-0.8036$$

(3) $$\text{Distance}[m]=599.3{\left(\frac{\text{Pixels}}{\text{Area}}\right)}^{-0.5138}-0.006038$$

Since the quadrotor is equipped with low-cost general-purpose cameras, high distortion is observed when taking images. Fortunately, they can be characterized and corrected using a calibration and mapping procedure. In order to correct the distortion, it is necessary to take into account the radial and tangential factors. The presence of the radial distortion manifests in the form of the “barrel” or “fish-eye” effect, while tangential distortion occurs because the lenses are not perfectly parallel to the image plane.

In this work, we made use of the algorithms available from OpenCV libraries [16]. Currently, OpenCV supports three types of objects for calibration: asymmetrical circle pattern, a symmetrical circle pattern and a classical black-white chessboard. The method used for the elimination of the optical distortion on the images from the frontal camera of the AR.Drone 2.0 was the chessboard method. The procedure consists of taking snapshots of this pattern from different points of view (POV) of the chessboard; the algorithm implemented detects the corners and the intersections on the chessboard and creates an equation. To solve the equation, it is required to have a predetermined number of pattern snapshots to form a well-posed equation system.

In practice, due to the amount of noise present in our input images, good results were obtained using 10 snapshots of the input pattern from different positions. After solving the equation system, the parameters of the correction matrix are obtained and output as XML/YAML files.

**Figure 4:**
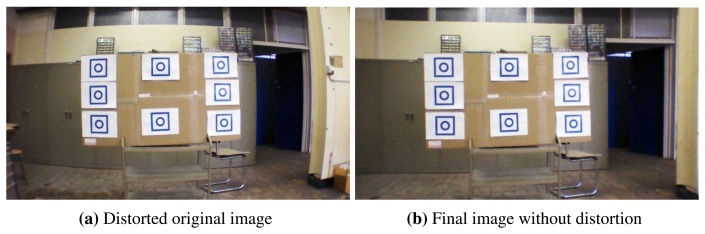
AR.Drone 2.0 pictures from the frontal camera: (**a**) original image with radial distortion; (**b**) image obtained after the remap process with the calculated distortion parameters.

This calibration experiment needs to be carried out only once. Then, inside the main application, once the files are loaded, a mapping function from OpenCV libraries is executed to eliminate the camera distortion. Finally, the distortion of the original image (Figure 4a) is eliminated as depicted in Figure 4b. Although a small part of the information is removed during the image processing procedure, the image is distortion free afterward.

#### 2.2.4. Processing Unit and Communication Channels

Two main circuit boards compose the processing unit of the drone:

- The mother-board holds the 1-GHz 32-bit ARM Cortex A8 processor with 800-MHz video DSP TMS320DMC64X, running a Linux-based real-time operating system.
- The second board uses a 16-bit PIC micro-controller navigation board, which interfaces with the sensors at a frequency of 40 Hz.

Regarding communication, there are four main services to connect with the AR.Drone:

Control and configuration of the drone is realized by sending AT commands on UDP port 5556. The transmission latency of the control commands is critical to the user experience. Those commands are to be sent on a regular basis (usually 30-times per second).

Information about the drone (like its status, its position, speed, engine rotation speed, *etc.*), called navdata, are sent by the drone to its client on UDP port 5554. These navdata also include tag detection information that can be used to create augmented reality games. They are sent approximately 30-times per second.

A video stream is sent by the AR.Drone to the client device on port 5555 with the TCP protocol. Given this protocol has a confirmation step, it presents a video streaming time delay of 360 ms, approximately. Image and video resolution can be selected between 360 p and 720 p. However, changing the video to 720 p creates a very noticeable lag between real time and the video. There was about a two-second delay. Images from this video stream can be decoded using the codec included in this SDK. The embedded system uses a proprietary video stream format, based on a simplified version of the H.263 UVLC (Universal Variable Length Code) format.

A fourth communication channel, called the control port, can be established on TCP port 5559 to transfer critical data, in opposition to the other data that can be lost with no dangerous effect. It is used to retrieve configuration data and to acknowledge important information, such as the configuration.

### 2.3. Position Estimation Using Sensor Fusion

Depending on the application, there are two possibilities to estimate the position of the quadrotor: (1) from camera images to situate the quadrotor on the *X, Y* plane; and (2) the AR.Drone provides an estimation of the translational speeds by using its on-board sensors and an optical flow algorithm, making it also possible to estimate the position by integrating the mentioned speeds.

**Figure 5:**
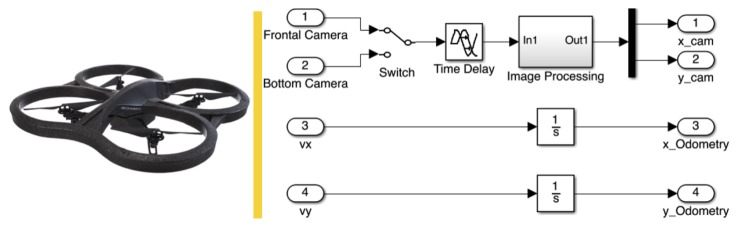
Sensors used for position estimation of the AR.Drone in the *x, y* plane.

Figure 5 describes the sensor's possibilities to estimate the position on the (*X, Y*) plane, each having positive and negative characteristics. On the one hand, odometry allows position estimation with almost no delay, but it suffers from drifting, producing an error that increases with time. On the other hand, to compute the position estimation based on optical measurements requires the use of patterns located in known positions, introducing also the problem of the additional time delays and noisy signals due to varying light conditions. An additional obvious difficulty is that once the pattern is out of the image, it is not possible to estimate the position.

The solution to achieve a reliable position estimation consists of combining the information from the two sensors. In order to reduce the drift effect and noise, odometry is used to read the variations and the optical sensor to find an offset-free measurement. The simplest and functional combination consists of using the optical sensor only when the standard deviation of the last five samples obtained from odometry is bigger than a tolerance value.

A time delay of about 100 ms is present due to latency in the wireless communication. However, the video signal has an additional time delay present in the video channel, which is directly related to the amount of data to be sent. For example, higher camera resolution introduces larger delays (*i.e.*, 330 ms approximately for an image of 640 × 360 pixels).

The position obtained from the camera represents the offset-free position, but “n” samples before, where “n” represents the time delay in samples (*i.e.*, *n* = 5 with *T_s_* = 66 (ms)). Next, it is possible to integrate the speed values of the last five samples, in order to obtain the position estimation from odometry up to time “n−1”. Equation (4) describes the method presented to eliminate the time delay effect on the video signal using a combination with odometry, assuming the dead time is a multiple of the sample time.

(4) $$x_{\left(k\right)}=x_{\text{cam}}\left(N_{d}\right)+T_{s}\sum_{k=-\left(N_{d}-1\right)}^{k=0}v_{x\left(k\right)}$$

where *x* is the final position in meters, *N_d_* = *T_d_*/*T_s_*, and *T_s_* is the sample time: 66 ms; *k* represents the samples; *v_x_* is the speed on the *x* axis; and *x_cam_* represents the position obtained from the camera with a constant time delay *T_d_* = 330 ms.

**Figure 6:**
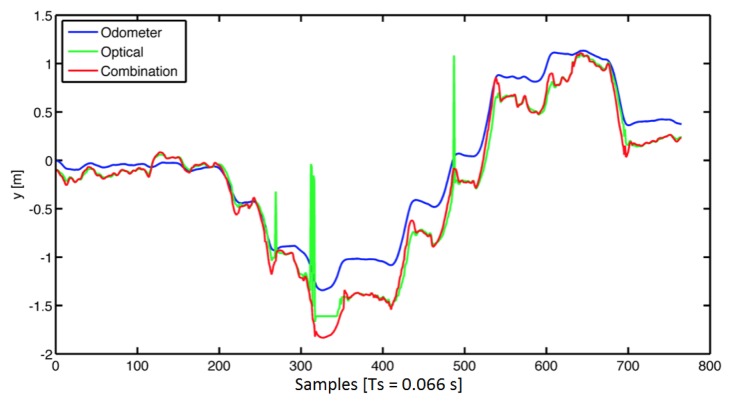
Position values in an open loop obtained from the image processing (green), the odometry (blue) and the fused response (red).

Figure 6 presents the performance of the estimation obtained, after using the proposed data fusion to correct the position measurements in the “*Y*” axis.

## 3. Quadrotor Dynamics and Identification

### 3.1. Coordinates System

**Figure 7:**
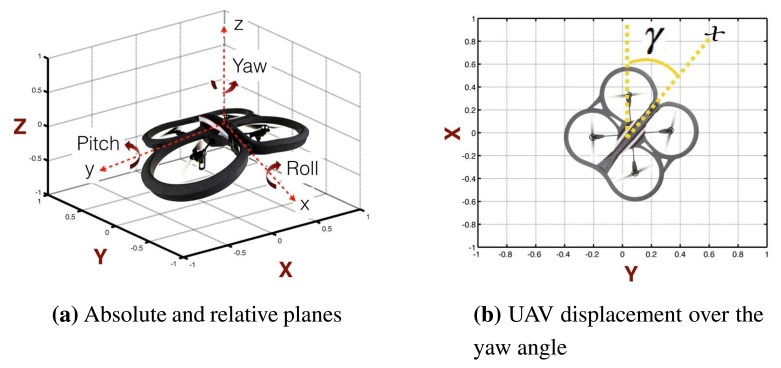
UAV axes: (**a**) difference between absolute axes (*X, Y, Z*) and relative axes (*x, y, z*); (**b**) UAV displacement on the (*x, y*) plane with respect to the absolute plane.

The quadrotor's aerial movements are similar to those of a conventional helicopter. The quadrotor has four degrees of freedom (DOF): rotation over pitch, roll and yaw and translational movements over *x*, *y* and *z*, as depicted in Figure 7a. Notice that through rotational movement along the transversal *y* axis (pitch), translational movement on the *x* axis is achieved. A similar conclusion can be drawn for rotation over roll and translational movement on *y*.

It is worth noting that the coordinate system described above (*x*, *y*, *z*), represents a relative coordinate system used by the internal controllers (low layer). Using such a coordinate system instead of absolute coordinates (e.g., *X*, *Y*, *Z*) in the high layer will yield errors. For example, notice that by rotating the quadrotor, the relative coordinates (*x, y*) will change with respect to the absolute coordinates, as depicted in Figure 7b.

**Figure 8:**
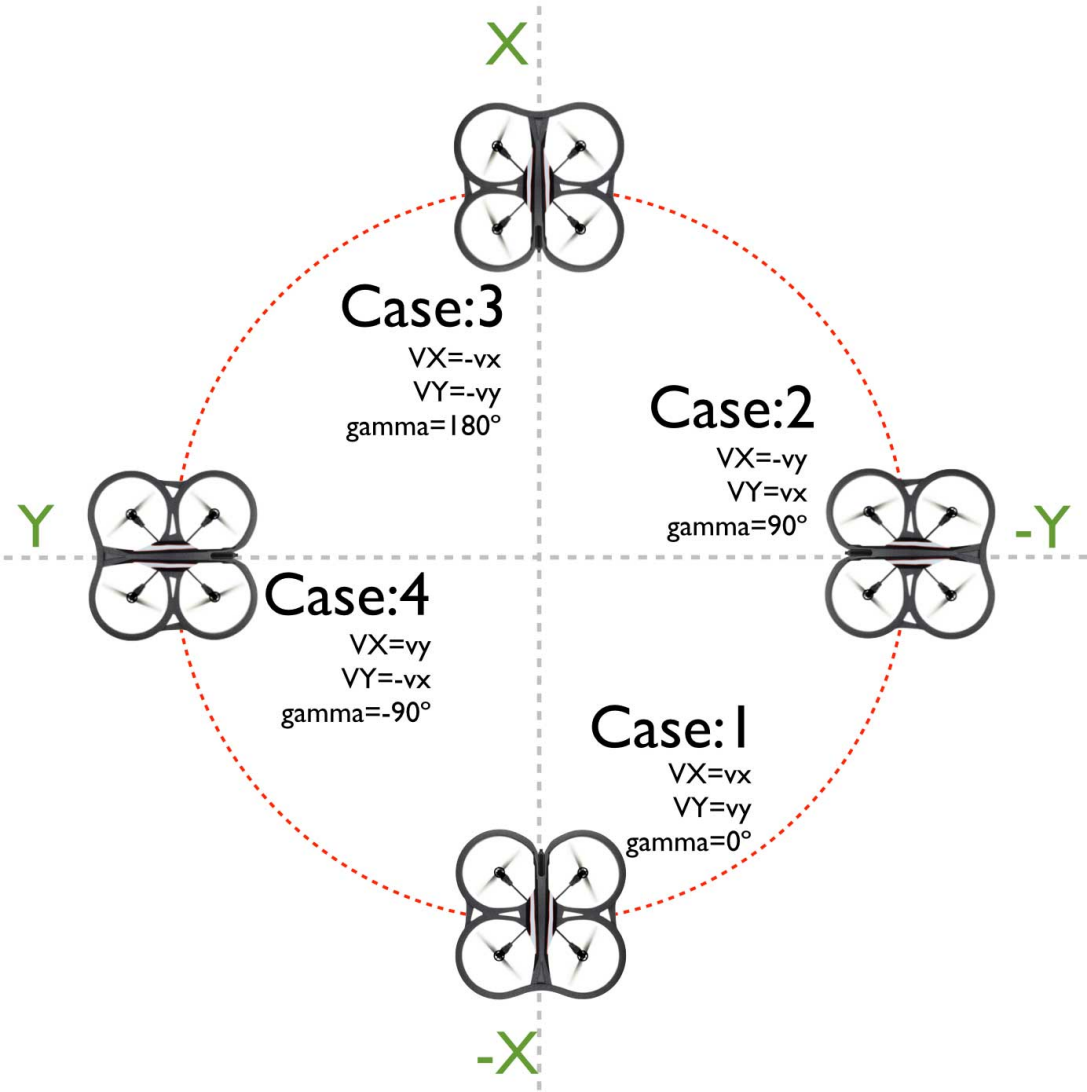
Case studies to describe the relation between the absolute and the relative coordinate systems.

Concerning the relationship between the relative and the absolute coordinate systems, four cases were analyzed (Figure 8). The first case represents a null angular displacement on the UAV orientation with respect to the absolute space, thus meaning that the speeds in the relative axes are the same as those of the absolute axes: “*V_X_* = *v_x_*” and “*V_Y_* = *v_y_*”. Cases 2 and 4 represent an angular displacement of 90° and −90°, respectively. The third case represents an angular displacement of −180°, which implies an opposite effect in the “*x*” and “*y*” axes.

Clearly, the relationship between the two coordinate systems is defined by the gamma (*γ*) angle, defined in Figure 7b. Consequently, equations describing the speeds of the UAV in the absolute system are defined as a function of the speed in the relative coordinates and (*γ*), as follows:

(5) $$V_{X}=v_{x}\cos(\gamma )-v_{y}\sin(\gamma )$$

(6) $$V_{Y}=v_{x}\sin(\gamma )-v_{y}\cos(\gamma )$$

After integrating the “*V_X_*” and “*V_Y_*” absolute speeds, it is possible to estimate the position of the UAV in the 3D space; this procedure is known as odometry. It is also important to note that Equation (6) depends on the yaw angle, which suffers from drifting over time, thus producing a “biased” estimation.

### 3.2. System Identification

Due to the internal control, the quadrotor behaves as a set of single-input single-output (SISO) systems, therefore making it possible to perform parametric identification on each degree of freedom. This is realized using the prediction error method [17] and a pseudo-random binary signal (PRBS) as the excitation signal. A sampling time of 5 ms for yaw and 66 ms for other degrees of freedom are chosen based on the analysis of dynamics performed in a previous work [18]. The transfer functions obtained are given by:

(7) $$\begin{array}{ll} \frac{v_{x}(s)}{v_{x}^{*}(s)} & =\frac{7.27}{\left(1.05s+1\right)}e^{-0.1s}\\ \frac{v_{y}(s)}{v_{y}^{*}(s)} & =\frac{7.27}{\left(1.05s+1\right)}e^{-0.1s}\\ \frac{v_{z}(s)}{v_{z}^{*}(s)} & =\frac{0.72}{\left(1.23s+1\right)}e^{-0.1s}\\ \frac{\dot{\gamma }(s)}{\dot{\gamma }(s)} & =\frac{2.94}{\left(0.031s+1\right)}e^{-0.1s} \end{array}$$

where *γ̇* is the angular speed in yaw. The time delay in Equation (7) represents the average time delay present due to the communication in the control channel, *i.e.*, the action on the motors is received approximately 100 milliseconds after it is set on the computer.

Notice that Equation (7) corresponds to the identification of the closed-loop system using information coming from the IMU board. The inputs are the setpoints for speed (*i.e.*, $v_{x}^{*}$, $v_{y}^{*}$, $v_{z}^{*}$, γ̇*, and the outputs are the response of the internal control to follow those setpoints (*i.e.*, *v_x_*, *v_y_*, *v_z_*, γ̇*. In other words, what is being identified is the closed-loop dynamics of the quadrotor for each degree of freedom.

## 4. Path-Following Predictive Control

A robust position controller of the quadrotor is required to follow either a set of way-points or a trajectory and to reject disturbances efficiently. Based on previous work [19,20], it has been found that model predictive control (MPC) fulfills the required specifications for tasks of tracking and positioning in 3D space [21]. In this section, the control structure and the implemented position controller using MPC is introduced.

### 4.1. EPSAC-MPC Algorithm

MPC refers to a family of control approaches, which makes explicit use of a process model to optimally obtain the control signal by minimizing an objective function [22]. In this contribution, the extended prediction self-adaptive control (EPSAC) approach to MPC is briefly described; for a more detailed description, the reader is referred to [23].

A typical set-up for the MPC optimization problems is as follows:

(8) $$\Delta U=\arg\underset{\Delta U\in {\mathbb{R}}^{N_{u}}}{\min}\sum_{k=N_{1}}^{N_{2}}{\left[r(t+k|t)-y(t+k|t)\right]}^{2}$$

for *k* = *N*_1_ … *N*_2_, where *N*_1_ and *N*_2_ are the minimum and maximum prediction horizons, Δ*U* is the optimal control action sequence, *N_u_* is the control horizon, *r*(*t* + *k*|*t*) is a future setpoint or reference sequence and *y*(*t* + *k*|*t*) is the prediction of the system output.

In EPSAC, the predicted values of the output are given by:

(9) y(t+k|t)=x(t+k|t)+n(t+k|t)

Then, it follows that *x*(*t* + *k*|*t*) *is* obtained by recursion of the process model; using the control input *u*(*t* + *k*|*t*) and *n*(*t* + *k*|*t*) represents the prediction of the noise, which includes the effect of the disturbances and modeling errors.

A key element in EPSAC is the use of base and optimizing response concepts. The future response can then be expressed as:

(10) $$y(t+k|t)=y_{\text{base}}(t+k|t)+y_{\text{optimize}}(t+k|t)$$

The two contributing factors have the following origin:

- *y_base_*(*t* + *k*|*t*) is the effect of the past inputs, the *a priori* defined future base control sequence *u_base_*(*t* + *k*|*t*) and the predicted disturbance *n*(*t* + *k*|*t*).
- *y*_o_*_ptimize_*(*t* + *k*|*t*) is the effect of the additions *δu*(*t* + *k*|*t*) that are optimized and added to *u_base_*(*t* + *k*|*t*), according to *δu*(*t*+*k*|*t*) = *u*(*t*+*k*|*t*) − *u_base_*(*t + k|t*). The effect of these additions is the discrete time convolution of Δ*U* = {*δu*(*t*|*t*), …, *δu*(*t* + *N_u_* − 1|*t*)} with the impulse response coefficients of the system (G matrix), where *N_u_* is the chosen control horizon.

Once Equation (10) is obtained, then Equation (8) can be solved, thus obtaining the optimal control action that minimizes the cost function. At the next sampling instant, the whole procedure is repeated, taking into account the new measured outputs according to the receding horizon principle, thus introducing feedback into the control law.

### 4.2. Performance of Position Control

Using the identified model of the quadrotor Equation (7), the EPSAC controller is tuned to achieve the shortest settling time without overshoot. The tuning parameters for the proposed controller are presented in Table 1.

**Figure 9:**
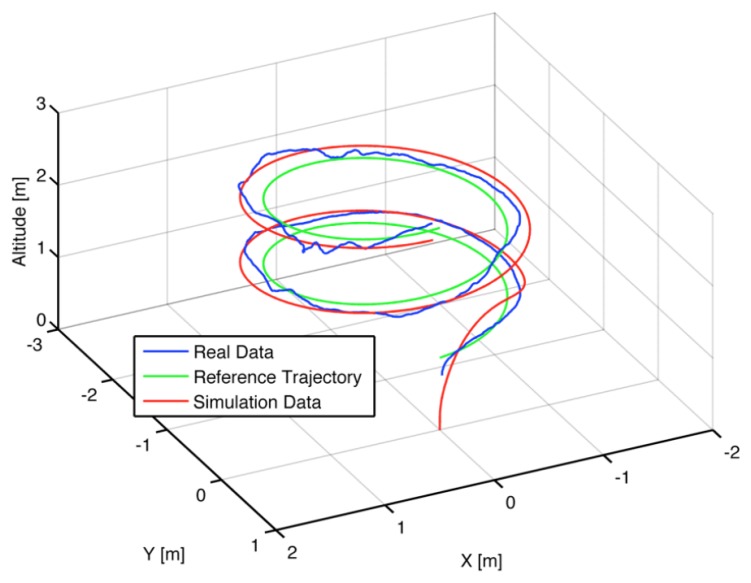
3D simulated and real response for path following of the AR.Drone 2.0 using EPSAC-MPC on (*X, Y* and *Z*) degrees of freedom.

Furthermore, the position controller is tested for path-following as depicted in Figure 9. It is observed that the controller is able to follow the trajectory with a small tracking error and almost no overshoot.

It is also important to highlight that the controllers are expected to work for the absolute coordinate system (*X*, *Y*); therefore, a transformation of the control action given by the MPC controllers to the relative coordinate system (*x*, *y*) is still necessary. This is performed based on Equations (5) and (6), thus determining the control actions as:

(11) $$v_{x}^{*}=V_{X}\cos(\gamma )+V_{Y}\sin(\gamma )$$

(12) $$v_{y}^{*}=-V_{X}\sin(\gamma )+V_{Y}\cos(\gamma )$$

where *V_X_* and *V_Y_* represent the absolute control action on the *X* and *Y* axis, respectively, at each sample time *T_s_.*

## 5. Application of Mapping for Precision Agriculture

In this study, we focus on improving the loading process in a forage harvester, by measuring the volume of grain inside a container in real time. This application lies in the field of mapping and precision agriculture.

### 5.1. Loading Process during Forage Harvesting

**Figure 10:**
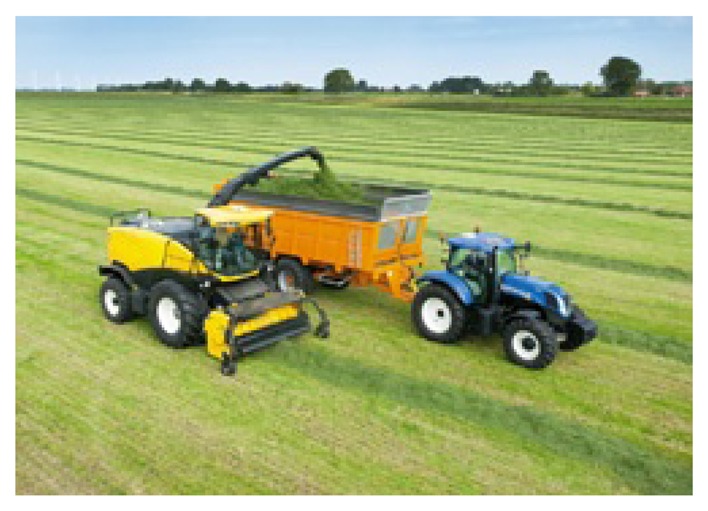
Combine harvester machine in a conventional loading process.

Corn silage is a high-quality popular forage for ruminant animals. During this process, the corn on the ground is extracted, chopped and then ensiled. Using combine harvesting machines for forage processes is a very common and demanding operation in agriculture. It requires two operators to operate the combine harvester and the tractor next to it. The forage is discharged from the harvester via a spout, where an orbital motor drives the spouts rotational movement. On the end of the spout, a flipper ensures that the forage is discharged accurately into the trailer. Figure 10 presents a combine harvester machine in a conventional loading process.

The success of the process depends on the two operators and their ability to maintain the correct position of the machines. Good synchrony allows one to reduce the loss of material and to optimize the trailer's space through efficient filling, thus ensuring an ideal flat material profile in the container. However, to achieve this synchronization, the harvester driver must focus on two tasks at once: (1) align the machine with respect to the tractor and the crop; and (2) adjust the actions to manipulate the spout with two degrees of freedom to send the material towards the container in the best way possible.

Automation of this process would enable operators to accurately drive the harvester while the system automatically fills the trailer, disregarding the distance or position of the two vehicles and even when visibility is limited. Consequently, the driver would benefit from improved operating comfort and safety, focusing only on the front of the machine, thus increasing the working rate.

In order to automate this process, the system must perform some special tasks: read arm angles, read the distance between the harvester and the trailer and make a 3D map of the material inside the trailer. In this work, we focus on producing a reliable 3D map of the volume of grain inside the trailer. This information can later be used directly for the operator or by a controller to guide the spout to the interest point of the lower height of the material to get a flat profile. Companies in the field have proposed several alternatives, which are briefly described in the next subsection.

### 5.2. Commercial Assistant Systems

Several alternatives (academic and commercial) have emerged to automate and improve the performance of the loading process. Here is included a short description for the three most representative solutions.

#### 5.2.1. Asuel

Asuel is prototype intended for position control of the spout of a forage harvester during the loading process [24]. The main objective is to build a mathematical model to estimate the volume and shape of the material inside the trailer. Estimation of the volume is corrected using information from Global Positioning Systems (GPS) and a model of the different actuators. The accuracy of the estimated profile is very low, given that it is difficult to obtain a good mathematical model, with the additional low resolution of GPS.

#### 5.2.2. Auto Fill

Autofill is a stereo camera-based spout control system [25] developed by Claas company. Using a fixed stereo camera has a significant advantage, allowing 3D perception compared to the traditional 2D images. When a trailer approaches the side of the forage harvester, the vision system detects its position. Once the trailer is detected, an overlay is drawn on the picture and shown to the driver. Then, a green line showing the estimated material level is drawn, thus engaging the AutoFill system. The system predicts where the crop jet will hit within the trailer using measurements of the spout and deflector rotations. Due to crop conditions and drift, the precision of the predicted hit point is not sufficient. Thus, the predicted jet trajectory is corrected online by measuring the distance to the jet. For this system, the main problem arises from the disturbance created from the dust created once the material falls inside the trailer.

#### 5.2.3. IntelliFill

Case New Holland (CNH) has recently launched a forage spout guidance called IntelliFill, which is based on a Time-Of-Flight (TOF) camera with 3D images. The optical sensor reads the light reflected back, obtaining a picture of the container filling. This camera allows the system to have functionality in the dark, as well as in bright day light [26]. The camera is mounted on the harvester's spout, and it can measure the distance to the four corners of the container and the filling volume inside the container. Through this information, the spout's turning angles can be automatically controlled.

### 5.3. Possible Limitation When Using a Fixed Camera

As described above, the goal is to reduce the loss of material and to optimize the trailer's space through an efficient filling, thus ensuring an ideal flat material profile in the container. This is achieved if operators maintain the correct position of the machines and if the operator of the harvester correctly manipulates the spout to send the material towards the container in the best possible way.

**Figure 11:**
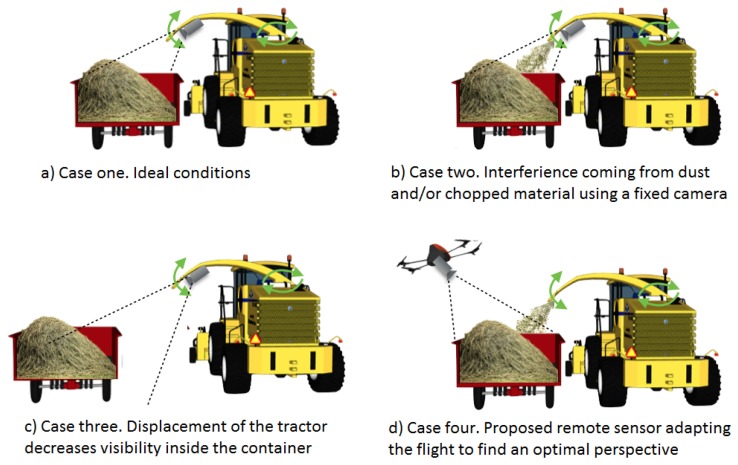
Possible limitations when using a fixed sensor and advantages of using a smart flying sensor for a loading application during forage harvesting.

Achieving a flat material profile inside the trailer is possible under good visibility conditions and flat terrains, with the additional help of a system to supervise the loading process as depicted in Figure 11a. Nevertheless, some limitations appear when placing the sensor (*i.e.*, camera) in the arm of the harvester machine. For example, noise in the images due to interference coming from dust, the small particles of chopped material and mechanical vibrations (Figure 11b) or a decrease of visibility due to an increase of the distance between the vehicles (Figure 11c). These difficulties can be diminished by using a flying sensor, because the camera can be placed in a better position, thus increasing visibility inside the trailer despite dust or a large distance between the vehicles (Figure 11d). Additional advantages can be obtained if other information is extracted from images during flight (e.g., density of the crop in front of the harvester to regulate the harvester speed properly) or if other sensors are installed.

### 5.4. Proposed Alternative by Using a UAV

**Figure 12:**
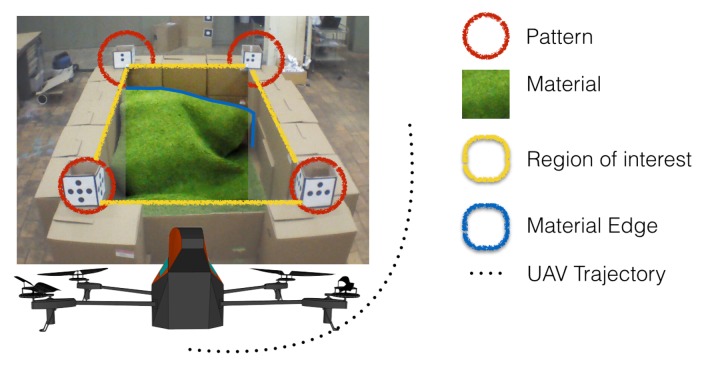
Experimental setup description.

A solution to the overloading problem could be the use of an UAV acting as a remote sensor, as depicted in Figure 11d. The quadrotor should follow the vehicles, read the profile disposition inside the container and, through image processing, detect the relative distance between the harvester and the trailer, *i.e.*, to minimize forage losses during the discharging process. A simple lab-scale system is utilized as proof-of-concept of the proposed solution. Figure 12 shows the setup platform used to emulate the tractor-trailer with the material. The emulated container has 2.0 × 1.5 × 1.0 m for length, width and height, respectively.

The UAV is in charge of collecting information from the process; in other words, it must follow the container and read the profile of the material. The patterns in the corners of the container are placed to provide references to the quadrotor and to delimit the region of interest. Although color patterns can be used, a higher robustness was observed with black and white patterns, given they have less variations for different light conditions.

## 6. Experimental Validation of the Proposed Smart Flying Sensor

### 6.1. Structure of the Proposed Solution

The experiment consists of flying with the quadrotor around the emulated container referenced by the patterns on the corners. Once the view is focused on a point of view (POV), the container is segmented by using triangulation with the pattern references. Subsequently, color segmentation can be applied to identify the material profile. Note that at least two pictures are needed to reconstruct a 3D surface representing the relief of the material.

**Figure 13:**
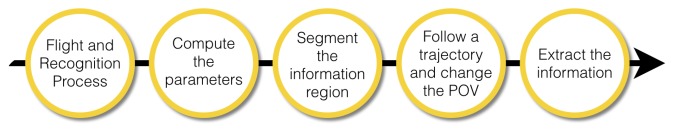
Methodology to build a 3D map of the material inside the trailer during a loading process using a forage harvester.

**Figure 14:**
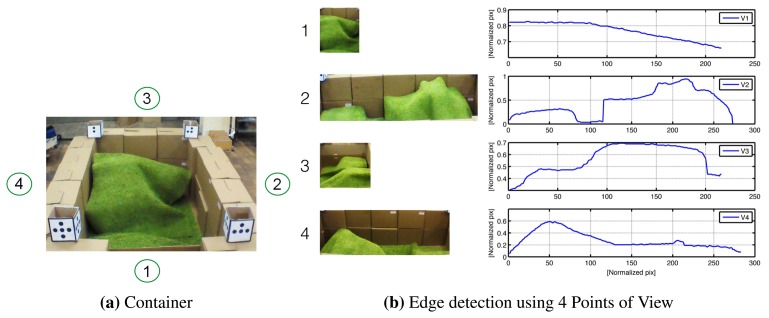
Example of (**a**) material profile in the container and (**b**) the information extracted using the edge detection algorithm considering four points of view (POV).

The complete procedure to build a 3D map of the material inside the container can be represented by the scheme in Figure 13. A description of the steps for the application are hereafter described:

Take-off and hold on: First of all, the quadrotor must be stabilized in space. Therefore, after the take-off, the quadrotor goes to an altitude setpoint of two meters (*i.e.*, z* = 2 m) and tries to hold the position.
Read the sensors: Once the quadrotor is hovering, the next step is to use the front camera and to take a picture in order to activate the image recognition process. It is important to mention that the camera is at about −45° with respect to the horizontal position. This is done in order to have a better view inside the container.
Reference the quadrotor: The distance between the UAV and the container is computed using information from the patterns. An image processing algorithm is in charge of computing the area inside the square of the pattern. Since this value is predefined and therefore known, the relative distance between the container and the UAV can be computed using Equation (3) and the time delay correction Equation (4). The controllers are designed to keep a fixed distance between the UAV and the container, centered on the container wall in a similar manner as depicted in Figure 12.
Read the area of interest: The controllers maintain the quadrotor at a fixed 3D point with respect to the container. When the position error is low and the angles (pitch and roll) are appropriate, the picture is reduced only to the inner container size Figure 14a. Here, color segmentation is used to extract the projection of the material on the walls by using edge detection techniques, as shown in Figure 14b.
Change the point of view (POV): In computer vision, the methods used to know depth information are based on the combination of images taken from different points of view (e.g., stereo vision). Given the flexibility of the UAVs, since a path-control algorithm using MPC has been implemented, it is possible to fly to other positions in order to get pictures from different points of view. The next step is thus to fly around the container pointing the camera inward, following a trajectory in four degrees of freedom in space, similar to the experiment performed in Figure 9, but using a constant altitude. The trajectory is calculated as a second order polynomial, knowing the actual coordinates and the destination point by using the translation between absolute and relative coordinate systems presented in Equations (11) and (12).
Extract the information: Once the pictures have been taken, a correction on the pictures was implemented to remove the “fish eye” effect present on the native photos. Then, the material is segmented, and the edge function is applied to calculate the contours of the material for each picture. These contours are in the form of vectors (e.g., V1, V2, V3 and V4) containing the information about the shape of the material on the container's wall. Figure 14 shows the material and its corresponding contour for four different points of view.

### 6.2. 3D Profile Computation

Image processing is composed of two main parts: the first part corresponds to the pre-processing (*i.e.*, acquisition, segmentation and classification), which is executed in OpenCV at the same sample time as in the UAV; the second part used to compute 3D map is executed in parallel in MATLAB with the process, but using a longer sampling time. Following MATLAB's notation, the surface obtained using four vectors (see Figure 14b) corresponding to the edge of the material inside the container is:

(13) $$S=0.25*[V_{1}(\text{end}:-1:1{)}^{\prime }*V_{2}+V_{1}(\text{end}:-1:1{)}^{\prime }*V_{4}+{V^{\prime }}_{3}*V_{2}+{V^{\prime }}_{3}V_{1}(\text{end}:-1:1)]$$

**Figure 15:**
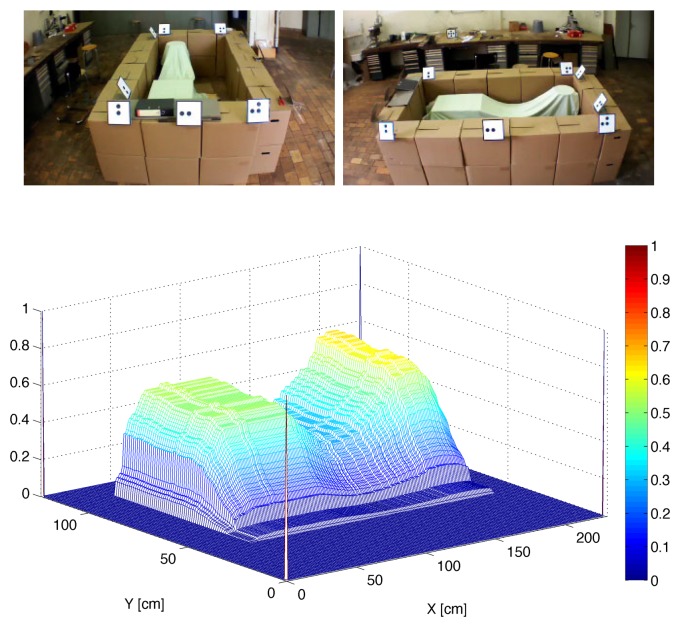
3D profile obtained experimentally using the smart flying sensor and two pictures.

A trade-off between accuracy and update time of the 3D map must be considered. Although using four images (four POV) has the advantage that a more accurate profile is obtained, this also implies a longer update time, since the UAV will require more time to take all pictures. A possible solution consists of using only two pictures, for which a good approximation of the 3D map can be computed, as depicted in Figure 15.

### 6.3. Performance of the Path-Following Controller

An important element for this application is the path-following controller, which is required to guarantee an accurate position of the quadrotor in the 3D space and to reject possible wind disturbances. Using the controller designed in Section 4, the quadrotor is able to automatically take-off and follow a pre-defined path around the container while taking the necessary number of pictures to compute the 3D profile.

**Figure 16:**
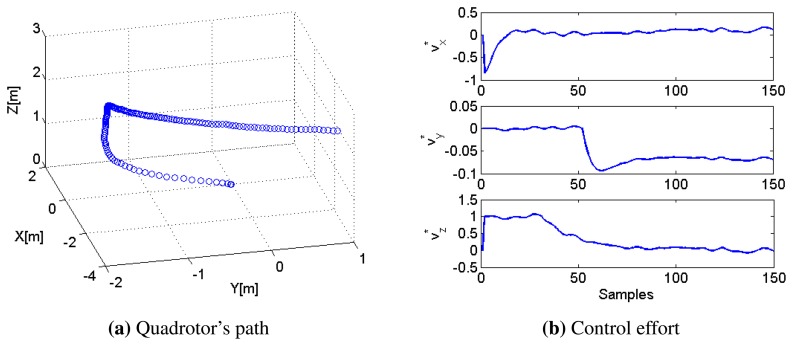
Performance of the path-following controller. (**a**) Quadrotor's path around the container in order to take two pictures and (**b**) control effort required by the MPC strategy.

The performance of the controller for the case of taking two pictures to approximate the material profile is depicted in Figure 16, including the final path followed by the UAV in Figure 16a and the control actions (i.e., setpoints to the low-layer internal controllers) required by the MPC in Figure 16b.

## 7. Conclusions

In this paper, we have presented the first steps towards the development of a smart flying sensor based on an unmanned aerial vehicle (UAV). The effectiveness of the proposed smart flying sensor is illustrated for the task of mapping the volume of grain inside a trailer during forage harvesting, using a lab-scale system.

The main achievements for this research are: (i) the obtained insight in the dynamics and coordinate systems of the low-cost quadrotor AR.Drone 2.0; (ii) the development of a position-estimation method with time delay compensation based on image processing and the inertial measurement unit (IMU); (iii) a method to build a 3D map using information obtained from a regular camera; and (iv) the design and implementation of a path-following control algorithm using MPC.

Future work includes incorporating a GPS for outdoor flight, together with the development of obstacle avoidance techniques to enhance the autonomy of the quadrotor. An extension to multiple UAVs and/or a combination with ground vehicles is also under investigation.

## Figures and Tables

**Table 1:** Design parameters for the extended prediction self-adaptive control (EPSAC) controllers. SISO, single-input single-output.

**SISO System**	***N*_1_**	***N*_2_**	***N****_u_*
*x, y*	1	15	1
*z*	1	30	1
*γ*	1	10	1

## References

[1] Sahota H., Kumar R., Kamal A., Huang J. An energy-efficient wireless sensor network for precision agriculture.

[2] Blackmore S. (1994). Precision Farming: An Introduction. J. Outlook Agric..

[3] Siuli Roy A., Bandyopadhyay S. Agro-sense: Precision agriculture using sensor-based wireless mesh networks.

[4] Berni J., Zarco-Tejada P.J., Suárez L., Fereres E. (2009). Thermal and narrowband multispectral remote sensing for vegetation monitoring from an unmanned aerial vehicle. IEEE Trans. Geosci. Remote Sens..

[5] Bouffard P. (2012). On-board Model Predictive Control of a Quadrotor Helicopter: Design, Implementation, and Experiments. Master's Thesis.

[6] Dullerud G. (2012). Modeling, Identification and Control of a Quad-Rotor Drone Using Low-Resolution Sensing. Master's Thesis.

[7] Dijkshoorn N., Visser A. An elevation map from a micro aerial vehicle for urban search and rescue.

[8] Mogenson M.N. (2012). The AR Drone LabVIEW Toolkit: A Software Framework for the Control of Low-Cost Quadrotor Aerial Robots. Master's Thesis.

[9] Ritz R., Muller M., Hehn M., D'Andrea R. Cooperative quadrocopter ball throwing and catching.

[10] Barrows G. Future visual microsensors for mini/micro-UAV applications.

[11] Clement A. (2013). Advances in Remote Sensing of Agriculture: Context Description, Existing Operational Monitoring Systems and Major Information Needs. Remote Sens..

[12] Pajares G. (2015). Overview and Current Status of Remote Sensing Applications Based on Unmanned Aerial Vehicles (UAVs). J. Photogramm. Eng. Remote Sens..

[13] Zarco-Tejada P., Hubbard N., Loudjani P. (2014). Precision Agriculture: An Opportunity for EU Farmers—Potential Support with the CAP 2014-2020.

[14] Mesas-Carrascosa F., Rumbao I., Berrocal J., Porras A. (2014). Positional quality assessment of orthophotos obtained from sensors onboard multi-rotor UAV platforms. Sensors.

[15] Bristeau P., Callou F., Vissiere D., Petit N. The Navigation and Control Technology inside the AR.Drone Micro UAV.

[16] OpenCV-Documentation. http://docs.opencv.org.

[17] Ljung L. (1999). System Identification: Theory for the User.

[18] Vlas T.E., Hernandez A., Copot C., Nascu I., de Keyser R. Identification and Path Following Control of an AR.Drone Quadrotor.

[19] Murcia H.F. (2014). A Quadrotor as Remote Sensor for Precision Farming: A Fill-Harvesting Case Study. Master's Thesis.

[20] Hernandez A., Copot C., Cerquera J., Murcia H., de Keyser R. Formation Control of UGVs Using an UAV as Remote Vision Sensor.

[21] Hernandez A., Murcia H.F., Copot C., De Keyser R. Model Predictive Path-Following Control of an AR.Drone Quadrotor.

[22] Camacho E.F., Bordons C. (2004). Model Predictive Control.

[23] De Keyser R. Model Based Predictive Control for Linear Systems. http://www.eolss.net/sample-chapters/c18/e6-43-16-01.pdf.

[24] Happich G., Harms H.H., Lang T. (2009). Loading of Agricultural Trailers Using a Model-Based Method. Agric. Eng. Int. CIGR J..

[25] Möller J. (2010). Computer Vision—A Versatile Technology in Automation of Agricultural Machinery. J. Agric. Eng..

[26] Posselius J., Foster C. Autonomous self-propelled units: What is ready today and to come in the near future.

